# Facilitating the removal of a foreign body in the tongue by intraoperative ultrasound guidance: A case report

**DOI:** 10.25122/jml-2023-0138

**Published:** 2023-07

**Authors:** Mostafa Alam, Ardeshir Tajbakhsh, Ashkan Badkoobeh, Mohammad Jahri, Mohsen Golkar, Anita Taheri, Milad Baseri

**Affiliations:** 1 Department of Oral and Maxillofacial Surgery, School of Dentistry, Shahid Beheshti University of Medical Sciences, Tehran, Iran; 2 Anesthesia Research Center, Shahid Beheshti University of Medical Sciences, Tehran, Iran; 3 Department of Oral and Maxillofacial Surgery, School of Dentistry, Qom University of Medical Sciences, Qom, Iran; 4 Dental Research Center, Research Institute of Dental Sciences, School of Dentistry, Shahid Beheshti University of Medical Sciences, Tehran, Iran; 5 School of Dentistry, Shahid Beheshti University of Medical Sciences, Tehran, Iran

**Keywords:** intraoperative ultrasound, foreign body, bur fracture, soft tissue trauma

## Abstract

The presence of soft tissue foreign bodies (FBs) presents a substantial concern due to their potential to induce both acute and chronic pain as well as tissue irritation. This case report documents the admission of a 25-year-old female with a history of bur fractures during endodontic treatment, accompanied by signs of infection. The clinical examination and radiographic assessment revealed an embedded foreign body within her tongue. The surgical procedure was informed by repeated ultrasound scans through the incision, facilitating precise targeting. Intraoperative ultrasound enables the accurate detection of submucosal foreign bodies in dynamic tissues like the tongue and facilitates focused and image-guided dissection, thereby decreasing surgical trauma to the delicate soft tissues.

## INTRODUCTION

Soft-tissue foreign bodies (FB) can be a significant problem due to their potential to cause acute and chronic pain and tissue irritation. Failure of FB removal probably leads to severe complications such as allergies, inflammation, or infection [1]. Radiographs are routinely used in surgical practice to identify radiopaque FBs [2, 3], while fluoroscopy has also been used to remove them [4]. A recent systematic review [5] has demonstrated that ultrasonography (US) [6] effectively detects FBs in distal extremities, locates them before surgery, and confirms their complete removal post-operation.

The surgeon should attempt to remove broken or damaged dental devices, gauze, and other medical materials that require removal during surgery. The body might also experience infection-related symptoms if some of these particles are left in the body, such as swelling and pain [7].

This case report illustrates the diagnostic challenges and surgical management of a patient with an embedded dental instrument fragment in the tongue, emphasizing the invaluable role of intraoperative ultrasound.

## CASE REPORT

**Figure 1:**
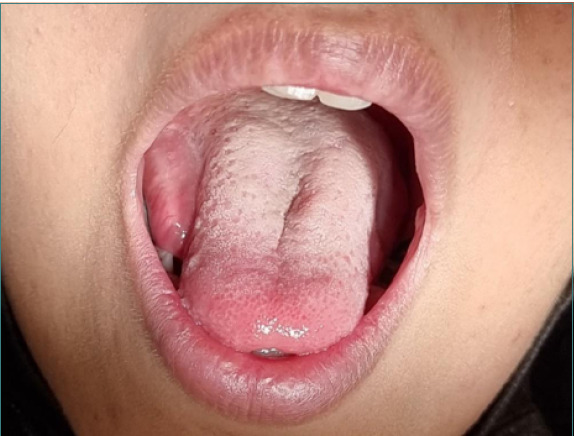
Localized swelling in the ventral surface of the patient’s tongue due to a FB

A 25-year-old female was admitted to Imam Hossein Hospital, presenting with persistent pain and the sensation of a foreign body localized in her tongue (Figure 1). She claimed that about two months ago, during the root canal therapy of the lower right first molar, the bur was suddenly broken, and her symptoms started two days after that appointment. The symptoms included swelling, irritation, pain, and pus formation, which were persistent for the previous two months. However, some symptoms, such as odynophagia, were relieved during this period. The patient was referred to the hospital by an oral medicine specialist with the diagnosis of FB in the tongue. The patient had two C-sections 5 and 3 years before and stated an allergy to penicillin. There were no indications of smoking or alcohol consumption.

**Figure 2:**
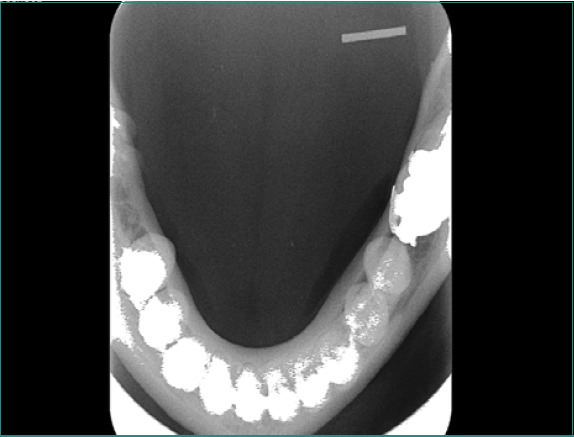
The occlusal view demonstrates a radiopaque area in the tongue

Clinical examination revealed a localized swelling, measuring 10mm x 30mm, on the right side of the tongue's ventral surface, characterized by firm consistency, ill-defined borders, tenderness, and pus formation. An occlusal view demonstrated a linear, high-density FB embedded in the tongue, deep to the ventral surface in posterolateral orientation (Figure 2).

**Figure 3:**
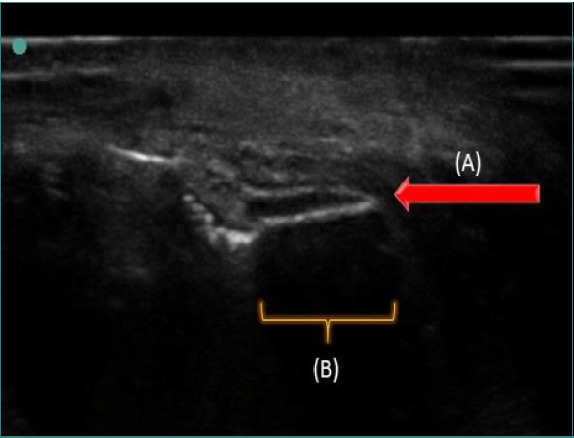
Intraoperative ultrasound. A: Foreign body as echogenic line, B: Posterior acoustic shadowing

General anesthesia was planned using US guidance because the fragment was deeply embedded. An intubation was performed with a Molt gag, and silk sutures were used to retract the tongue after nasal intubation. A linear transducer (HFL38 13-6MHz) (Sonosite S-Nerve) was used to locate the FB. Accurate scanning was performed by holding the tip of the tongue with one hand and compressing the body with the other. As the foreign body (FB) was linear and metal, we were specifically searching for an echogenic line with posterior acoustic shadowing to identify it. We also tried to place the probe in the longitudinal axis of the FB to thoroughly visualize it (Figure 3 A-B).

**Figure 4:**
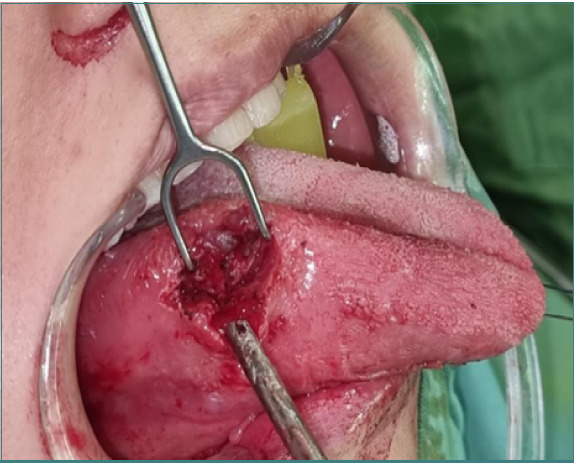
An incision was made to visualize the fragment

**Figure 5:**
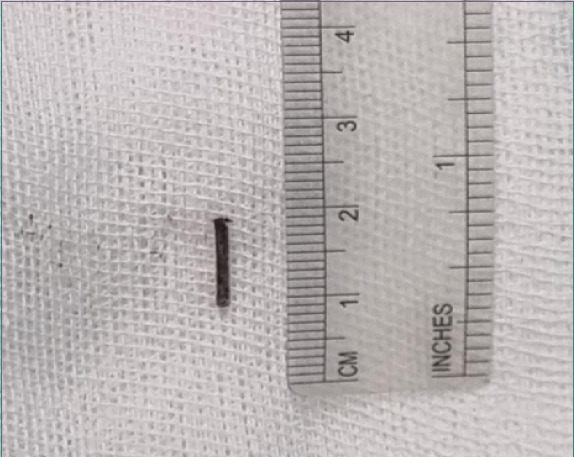
Measurement of the extracted fragment

A longitudinal incision was made (Figure 4), and the fragment was quickly located using repeated ultrasound scanning. The fragment was quickly located and removed with mosquito forceps (Figure 5). The incision was then closed with a silk 3/0 cutting suture.

**Figure 6:**
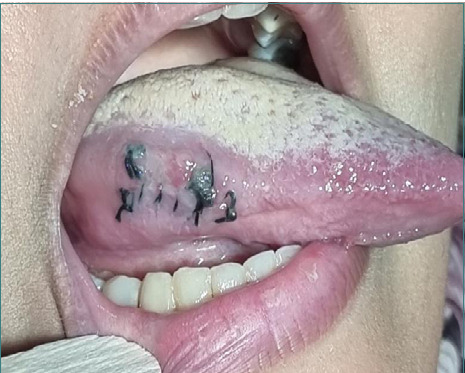
Suture line after seven days

**Figure 7:**
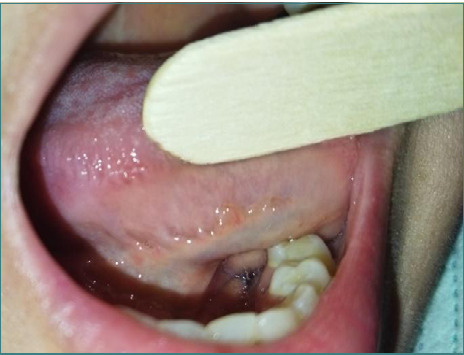
Suture line after six months, complete healing, and no signs of inflammation are evident

The patient was observed for 48 hours with intravenous antibiotics before being discharged. A seven-day follow-up revealed that the wound healed well, and the tongue exhibited no sensory or functional deficits (Figure 6). A 6-month follow-up confirmed complete healing signs at the incision site (Figure 7).

## DISCUSSION

The breaking of dental burs and endodontic files can occur for various reasons, including stress, device failure, fatigue, improper handling, and rust. Identifying foreign bodies through clinical examination alone may be challenging, especially when pain, swelling, and hematoma are present following an injury; it has been reported that up to 38% of FBs may remain unidentified in such cases [8].

To detect FBs and their precise location, imaging techniques are essential. Primary diagnosis of FB is conducted by simple radiographs. However, ultrasound has emerged as an effective option to diagnose soft tissue FBs with greater precision, both preoperatively and intraoperatively. Soft tissue FBs can be identified by ultrasound with high specificity and moderate sensitivity [5]. The high spatial resolution of ultrasound devices enables the detection of even millimeter-sized FBs, although operator experience can limit its effectiveness in visualizing superficial FBs [9]. It is possible to identify FBs by assessing their morphology and volumetric characteristics and determining their spatial location in three dimensions using high-frequency ultrasound probes (7-17 MHz) [1]. FBs are usually presented as a hyperechoic area with posterior acoustic shadowing or echo. A hypoechogenic halo can surround an FB long after a traumatic event caused by granulomatous inflammation [8, 9].

There is a need to distinguish between open wounds and wounds with a small entry hole when treating patients with diagnosed or suspected FBs retained in the soft tissues. In the case of open wounds, it is vital to investigate the lesion thoroughly. An excellent alternative to surgery for removing FBs retained in soft tissue is ultrasound-guided removal, which is inexpensive, repeatable, and carries a low risk of complications. Its entry point is usually less than one centimeter, and the scar tissue is small, so it does not have the same aesthetic effect as surgically removing FBs [8].

Recent studies have explored the use of ultrasonography to detect tongue edema associated with laryngoscope pressure [10] and tonsillar retractor use [11]. While otorhinolaryngologists commonly use US during diagnostics, intraoperative use is rare. Previous reports of intraoperative US for removing fishbones from the tongue have shown success, even when used as a second-line method after open-tongue exploration failed [6, 12]. By performing intraoperative US serially, embedded FBs can be located more accurately, which prevents more extensive tissue damage than blind exploration can. Focused surgery reduces tissue dissection, reducing the risk of injury to the lingual nerve and giving a faster recovery and better long-term function. In addition, it reduces postoperative pain.

## CONCLUSION

Fragmentation of dental instruments embedded in soft tissue poses minor challenges in identifying and removing foreign bodies (FBs). Intraoperative ultrasound, though underutilized, offers rapid localization of FBs by an experienced radiologist, promoting quicker recovery by minimizing dissection and trauma.

## Data Availability

Further data is available from the corresponding author on reasonable request.
